# Computational Investigation of BMAA and Its Carbamate Adducts as Potential GluR2 Modulators

**DOI:** 10.1021/acs.jcim.3c01195

**Published:** 2024-06-18

**Authors:** Isidora Diakogiannaki, Michail Papadourakis, Vasileia Spyridaki, Zoe Cournia, Andreas Koutselos

**Affiliations:** † Biomedical Research Foundation, Academy of Athens, 4 Soranou Ephessiou, Athens 11527, Greece; ‡ Department of Chemistry, Physical Chemistry Laboratory, National and Kapodistrian University of Athens, Panepistimiopolis, Athens 15771, Greece; § Department of Nursing, Faculty of Health Sciences, Hellenic Mediterranean University, Heraklion, Crete 71004, Greece; ∥ School of Chemical Engineering, National Technical University of Athens, Heroon Polytechniou 9, Zografou 15780, Greece

## Abstract

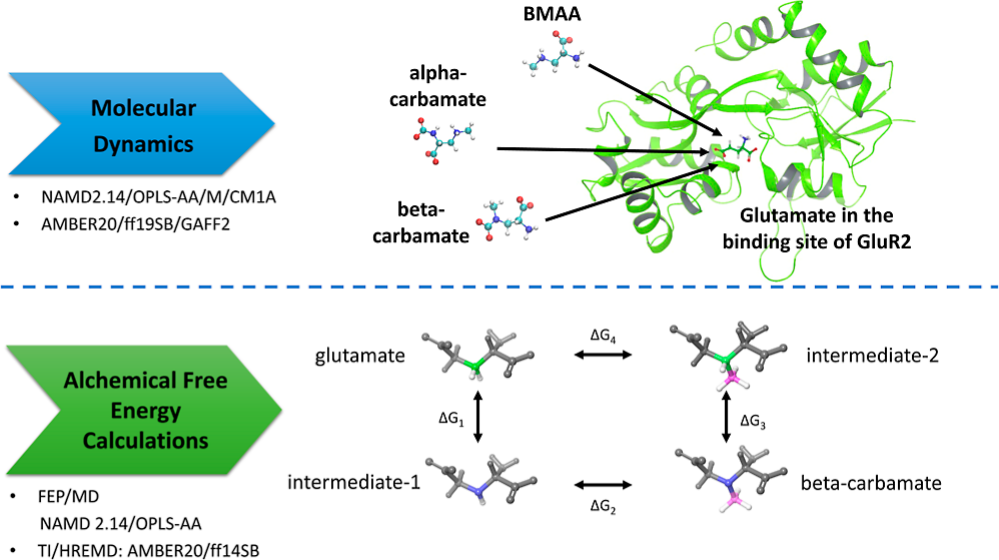

Beta-*N*-methylamino-l-alanine (BMAA) is a potential neurotoxic nonprotein amino acid, which can reach the human body through the food chain. When BMAA interacts with bicarbonate in the human body, carbamate adducts are produced, which share a high structural similarity with the neurotransmitter glutamate. It is believed that BMAA and its l-carbamate adducts bind in the glutamate binding site of ionotropic glutamate receptor 2 (GluR2). Chronic exposure to BMAA and its adducts could cause neurological illness such as neurodegenerative diseases. However, the mechanism of BMAA action and its carbamate adducts bound to GluR2 has not yet been elucidated. Here, we investigate the binding modes and the affinity of BMAA and its carbamate adducts to GluR2 in comparison to the natural agonist, glutamate, to understand whether these can act as GluR2 modulators. Initially, we perform molecular dynamics simulations of BMAA and its carbamate adducts bound to GluR2 to examine the stability of the ligands in the S1/S2 ligand-binding core of the receptor. In addition, we utilize alchemical free energy calculations to compute the difference in the free energy of binding of the beta-carbamate adduct of BMAA to GluR2 compared to that of glutamate. Our findings indicate that carbamate adducts of BMAA and glutamate remain stable in the binding site of the GluR2 compared to BMAA. Additionally, alchemical free energy results reveal that glutamate and the beta-carbamate adduct of BMAA have comparable binding affinity to the GluR2. These results provide a rationale that BMAA carbamate adducts may be, in fact, the modulators of GluR2 and not BMAA itself.

## Introduction

Beta-*N*-methylamino-l-alanine (BMAA) is a natural, neurotoxic nonprotein amino acid that is produced by a range of ecologically diverse phytoplankton groups such as cyanobacteria, diatoms, and dinoflagellates.^1^ Such cyanobacteria live in the roots of the cycad tree, whose seeds are a common food source consumed by humans; thus BMAA may end up in the human body through the food chain. In addition, BMAA may reach the human body through the food chain in aquatic systems as BMAA can be transferred from cyanobacteria via zooplankton to organisms at higher trophic levels.^2^ It has been established that BMAA has neurotoxic and neuro-excitatory properties and that is a source of neurodegenerative disorders in humans; specifically, BMAA has been detected in post-mortem brain and spinal cord tissues of amyotrophic lateral sclerosis, Parkinson’s, and Alzheimer’s patients.^3^

**Figure 1:**
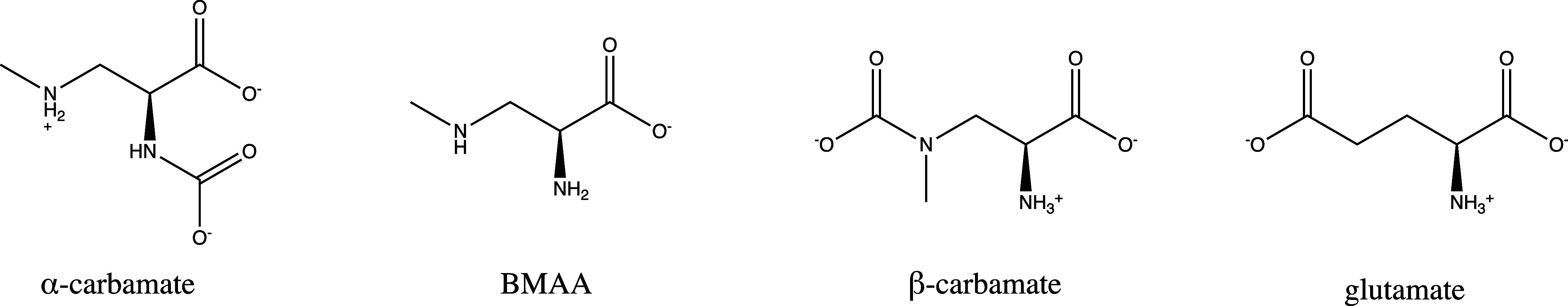
Structures of alpha-carbamate, BMAA, beta-carbamate (l-isomers), and the natural agonist glutamate.

Neurotoxicity of BMAA is dependent on the presence of bicarbonate, which is produced from the interaction of carbon dioxide with water.^3^ BMAA is nontoxic in a physiological salt solution, but in the presence of bicarbonate, which is present in nature and the human body, a carbamylation reaction takes place that produces BMAA carbamate adducts such as alpha-carbamate and beta-carbamate in a ratio of 86:14, respectively.^3^ BMAA and its carbamate adducts share a high structural similarity with the natural agonist glutamate (Figure 1).^4^ Glutamate and structurally similar substances activate several glutamate receptors (GluRs), which act as neurotransmitters in the nervous system.^5^ GluRs are divided into two subcategories, the metabotropic receptors and the ionotropic receptors (iGluR) that include kainate, α-amino-3-hydroxy-5-methyl-4-isoxazolepropionic acid (AMPA), and *N*-methyl-d-aspartate (NMDA).^6,7^ The activation of iGluRs is important in the development and function of the nervous system, while they are also essential in memory and learning. Dysfunction of iGluRs leads to excitotoxic cell death.^6^ It has been therefore hypothesized that the role of BMAA in the onset and progression of neurodegenerative diseases might be linked to the dysfunction of iGluRs, as BMAA and its carbamate adducts might bind in the binding site of iGluRs, due to their structural resemblance with glutamate.^8,9^

A well-studied subtype of AMPA receptors that may be influenced from the binding of BMAA and its carbamate adducts is the ionotropic glutamate receptor 2 (GluR2).^10^ The binding of glutamate to GluR2 has been examined extensively with both experimental and computational methods. Crystal structures of glutamate bound to GluR2 (PDB ID: 3DP6,^11^1FTJ^12^) reveal that the binding pocket for glutamate is situated between the S1 and S2 domains (ligand binding core).^6,12,13^ The IC_50_ for the displacement of 3H-AMPA by glutamate has been estimated to be 821 nM.^12^ The concentration of the substrate AMPA in these experiments was only 20 nM, which can be considered much lower than Km. Because IC_50_ values approximate Ki when the concentration used in the assay is much lower than K_m_, we can approximate the glutamate free energy of binding to the GluR2 to be −8.4 kcal/mol (821 nM at 310 K).^12^ The binding free energy of glutamate in the closed conformation of GluR2 was also assessed computationally by Speranskiy and Kurnikova^63^ using the MD/PBSA method and resulting in an estimated value of approximately −13 kcal/mol. Furthermore, Mamonova et al.^64^ utilized umbrella sampling and molecular dynamics (MD) simulations to describe how the GluR2 ligand binding core interacts with glutamate as well as the conformational changes upon ligand binding. One simplified representation of this process envisioned the protein resembling a clamshell, which encloses glutamate and seals the cleft formed by two lobes connected by a hinge.^64^ Taking into account the protein reorganization energy, as determined in this study to be approximately −4 kcal/mol, along with the previously computed binding free energy of glutamate in the closed conformation of GluR2, an estimation of the total binding energy between glutamate and the GluR2 S1S2 ligand binding core was computed to be −9 kcal/mol. In addition, the standard binding free energy of glutamate (along with four other ligands) to the GluR2 has also been calculated using free energy perturbation calculations (FEP) coupled with MD simulations.^16^ The standard binding free energy for glutamate to S1/S2 GluR2 using PDB ID: 1FTJ has been estimated to be −8.5 ± 1.8 kcal/mol, in agreement with experimental results from competition assays with 3H-AMPA (−8.4 kcal/mol, see discussion above).^14,16^ Finally, atomistic MD simulations have also been employed to investigate the mechanism by which glutamate binds to GluR2.^15^ Two possible pathways with which glutamate traverses during ligand binding have been identified using the string method, and the overall free energy difference between the initial unbound state (protein in the apo state, PDB ID 1FTO(12)) and the final bound state in pathways 1 and 2 has been estimated to be −5.8 and −8.8 kcal/mol, respectively. The pathway with the lowest free energy of binding corresponds to the glutamate binding pose with PDB ID 1FTJ, which is the structure that we also used in our relative binding free energy (RBFE) calculations herein.

While studies suggest that the toxic activity of BMAA is linked to the binding of BMAA and its carbamate adducts to the GluR2 glutamate binding site, to the best of our knowledge, there are no previous computational or experimental studies on the binding of these molecules to GluR2, and their mechanism of binding and energetics have not been yet elucidated.^8,9^ Therefore, we set out to investigate the stability and binding affinity of BMAA and its carbamate adducts (l-isomers) to GluR2 in comparison to the natural agonist, glutamate, to assess whether these molecules could bind on GluR2. If their affinity to GluR2 is similar to or stronger than glutamate affinity, BMAA and its carbamate adducts could potentially provoke dysfunction in neurons, leading to neurodegenerative diseases through binding to the GluR2. Herein, we study the stability of BMAA and its adducts in GluR2 using atomistic MD simulations and RBFE calculations using two force fields to quantify the binding of the beta-carbamate adduct of BMAA with respect to glutamate. Our findings suggest that BMAA carbamate adducts may be in fact the modulators of GluR2 and not BMAA itself.

## Methods

### Ligand Structural Similarity and Selection of Crystal Structure for the Calculations

To examine the two-dimensional (2D) structural similarity of BMAA and its carbamate adducts with respect to the natural agonist glutamate, the Tanimoto coefficient^17^ (*T*_c_) of each ligand compared to glutamate was calculated using ChemBioServer 2.0^18^ (see the Supporting Information). In addition, we also computed the three-dimensional (3D) similarities between the compounds using the Shape Screening module provided by Schrödinger 2020.3 suite^19^ and the Pharmacophore type scoring function provided by Phase.^20,21^

**Figure 2:**
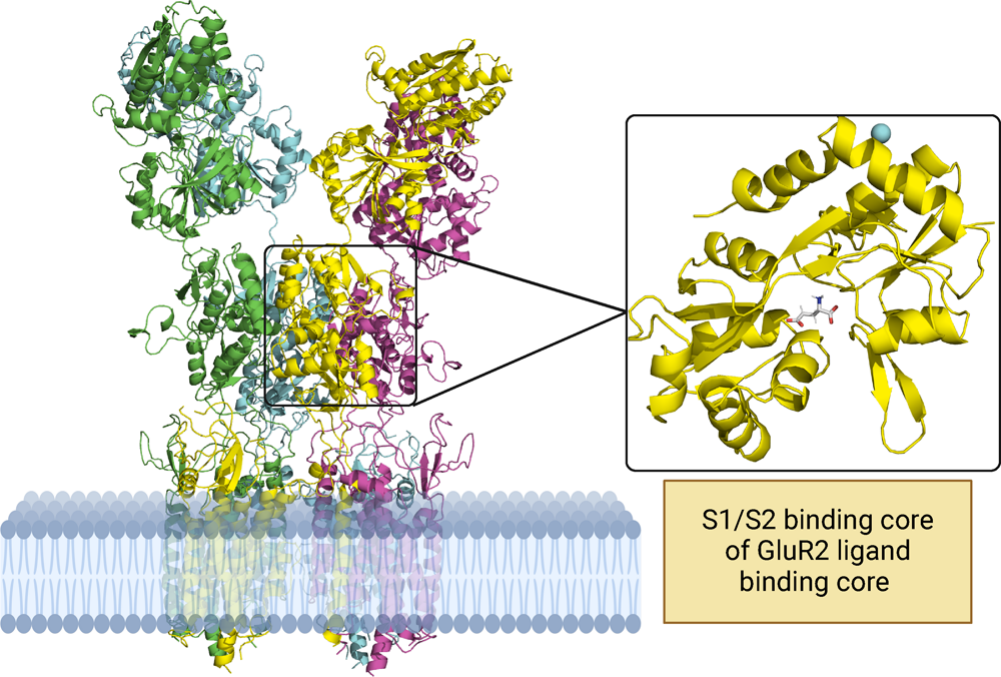
Structure of GluR2 embedded in a phospholipid bilayer. Inset: chain A of the S1/S2 ligand binding core of GluR2 (PDB ID 1FTJ) is illustrated together with the bound native agonist glutamate shown in stick representation.

The receptor crystal structure should be suitable for atomistic simulations of the protein–ligand complex;^22^ here, the glutamate-GluR2 complex structure was selected based on the following criteria: (1) resolution lower than 3.0 Å and (2) crystallization conditions close to body temperature (*T* = 310 K) and pH (pH = 7.4) (see also the Supporting Information). The crystal structure with PDB ID 1FTJ(12) meets the above criteria as it includes the S1/S2 ligand binding core of GluR2 in complex with glutamate and it is crystallized at 277 K, pH = 6.5, and resolution = 1.9 Å. Moreover, it has been successfully used in recovering the energetics of glutamate binding using computational work, in excellent agreement with experiments (see the Introduction). Because the crystallized protein with PDB ID 1FTJ is derived from *Rattus norvegicus*, we compared the rat protein sequence with a human protein sequence using BLAST,^23^ which showed a 99.5% sequence similarity between the two proteins. The only amino acid change on the S1/S2 core is the G231R mutation that is located at ca. 17 Å from the center of mass of the native agonist, glutamate. We thus concluded that PDB ID 1FTJ^12^ is a suitable starting structure for performing MD/ alchemical free energy simulations with glutamate as a ligand. Chain A of the GluR2 ligand binding core was isolated from the full trimer to speed up the simulations (Figure 2). Full details of the model construction and protein modeling are presented in the Supporting Information.

### Docking

Before creating starting configurations for BMAA and its carbamate adducts binding on GluR2, we assessed the ability of Glide 6.7^24−26^ to reproduce the glutamate crystal structure pose in the binding pocket of GluR2. Self-docking of glutamate was performed using default parameters (Figure S1) of Glide 6.7. BMAA, alpha-carbamate, and beta-carbamate were also docked into GluR2 using default parameters of Glide 6.7 to generate initial positions for the MD simulations. Docking scores were calculated using the Glide SP scoring function.

### MD Simulations with NAMD2.14/OPLS-AA/M/CM1A

Three replicas for each system, namely, glutamate/GluR2, BMAA/GluR2, alpha-carbamate/GluR2, and beta-carbamate/GluR2, were simulated for 100 ns using the NAMD 2.14 software. For the replica simulations, all conditions were identical except for different initial velocities. For the protein, the OPLS-AA/M^27^ force field was used. Ligand parameters were retrieved from the LigParGen^28^ Web server (OPLS-AA/CM1A^29^ force field), and the TIP3P^30^ potential was used for water. The systems were solvated in a cubic box of 15 Å length in each dimension with 45,891 water molecules. Counter ions were added to neutralize the total charge. Long-range electrostatic interactions were treated using the particle-mesh Ewald (PME) summation method.^31^ A time step of 2 fs was used. The temperature was kept constant at 310 K using the Langevin thermostat^32^ with a time constant of 1 ps. The pressure was isotropically maintained at 1 atm using the Nosé–Hoover barostat.^33^ The barostat oscillation period was set to 100 fs and the barostat damping timescale to 50 fs. The nonbonded potential energy functions (electrostatic and van der Waals) were smoothly truncated between cutoff distances of 10–12 Å.

Prior to MD simulations, all structures were relaxed with 50,000 steps of energy minimization using steepest descent. Heating followed, where the temperature was incrementally increased from 10 to 310 K for 0.6 ns. Then, the systems were equilibrated in the *NPT* ensemble for 1 ns. Finally, unbiased MD simulations were carried out for 100 ns. To assess the stability of the ligands in the binding cavity of the GluR2, the RMSD of each ligand was calculated with respect to the initial frame using the RMSD *Trajectory tool* of VMD 1.9.3.^34^

### MD Simulations with AMBER20/ff19SB/GAFF2

The same procedure for model construction, equilibration, and MD production protocols was followed using the AMBER20^35^ software with the ff19SB^36^ force field for the protein and GAFF2^37^ parameters that use AM1-BCC^38^ charges for the ligands. The Monte Carlo barostat^39^ of AMBER20 was used, and the pressure was set to 1 atm. The number of steps between the performed volume change attempts was equal to 100.

### RBFE Calculations

**Figure 3:**
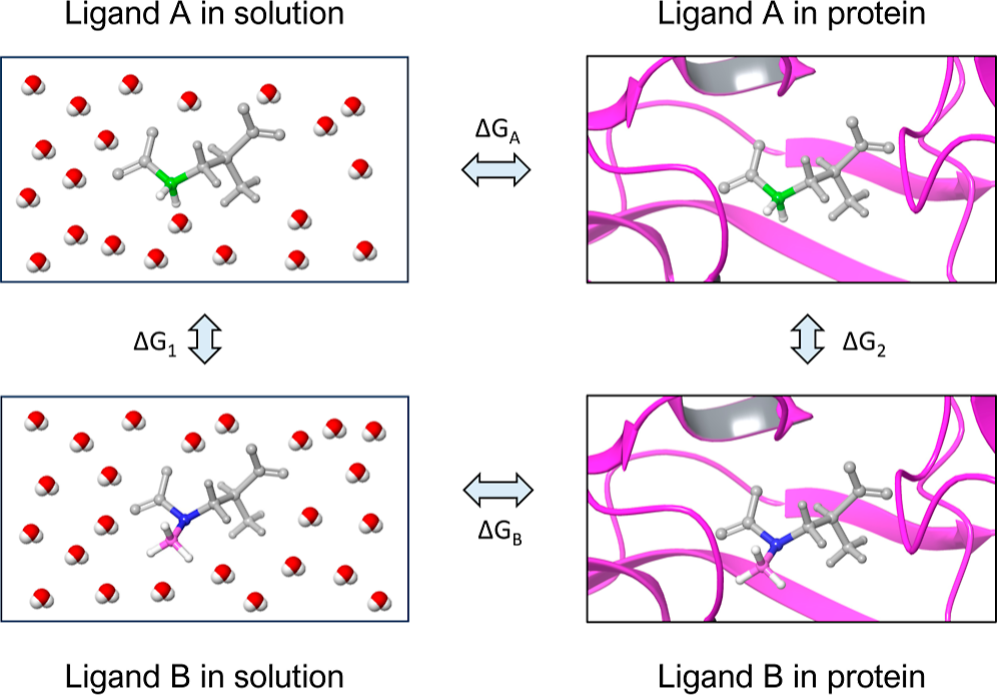
Thermodynamic cycle for the perturbation of ligand A (glutamate) to ligand B (beta-carbamate), in which a methylene bridge colored in green is replaced by a methylamino group colored in blue and pink. All other atoms are colored in gray. The relative binding free energy (ΔΔ*G*_bind_) of beta-carbamate with respect to glutamate can be calculated via two possible paths. Simulating the direct path (horizontal processes, Δ*G*_B_ – Δ*G*_A_) is slow to converge due to the large difference between the end states (solution versus protein environments). The alchemical path (vertical processes, Δ*G*_2_ – Δ*G*_1_) requires much smaller perturbations to the system and therefore tends to converge much faster. Here, ligand A (glutamate) is perturbed to ligand B (beta-carbamate) in the bound state (Δ*G*_2_) and the unbound state (Δ*G*_1_), the difference of which is identical to the direct path (due to the closed thermodynamic cycle). The double arrow for each process illustrates that both forward and backward perturbations are performed.

The RBFE of glutamate bound on GluR2 compared to beta-carbamate was computed with alchemical relative free energy calculations.^40−42^ An example of the thermodynamic cycle used for this study is illustrated in Figure 3. For more information on alchemical free energy theory, please refer to the Supporting Information or recent reviews.^41^

**Figure 4:**
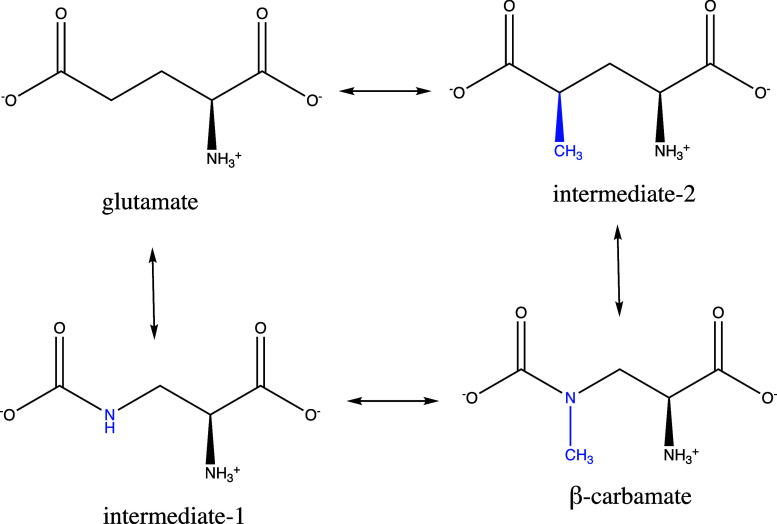
Perturbation network used for the RBFE calculations. Glutamate and beta-carbamate are connected through the use of two intermediate molecules to increase phase space overlap and create a closed cycle. The perturbed atoms with respect to glutamate are colored blue.

The perturbation between two ligands, in our case glutamate and beta-carbamate, is typically performed by connecting the two systems using a coupling parameter λ, such that the potential energy function U(λ) interpolates between the initial state (glutamate, λ = 0.0) and the final state (beta-carbamate, λ = 1.0). To increase the phase space overlap between the two ligands, we introduced two intermediate molecules, named intermediate-1 and intermediate-2 (Figure 4). In intermediate-1 a methylene bridge of glutamate is replaced by an amino group, and in intermediate-2 the nitrogen of the methylamino group of beta-carbamate is replaced by a carbon–hydrogen bond. In the generated RBFE network, each edge represents both forward and backward perturbations that are performed in both the bound and the unbound state. Moreover, a closed thermodynamic cycle is constructed, where hysteresis can be measured because the summation of the free energy change along each edge of the closed cycle should be zero.

The primary assumption of RBFE calculations is that all ligands in the congeneric series retain the same binding mode. Therefore, via Maestro Schrödinger Suite 2020–3,^19^ beta-carbamate, intermediate-1, and intermediate-2 were overlaid using, as a starting structure, the crystallographic binding orientation of glutamate into chain A of GluR2 (Figure S2).

### FEP/MD Simulations Using NAMD 2.14/OPLS-AA

For FEP/MD simulations using NAMD 2.14/OPLS-AA, we generated all input files using the FEPrepare Web server,^43^ which automates the setup procedure for relative binding FEP calculations for the dual-topology scheme of NAMD using OPLS-AA force field topology and parameter files. Then, all structures were equilibrated in the *NVT* and *NPT* ensembles for 1 and 2 ns, respectively. Production run simulations were run for 10 ns with NAMD of 2.14 in the *NPT* ensemble per λ window. The number of equidistant λ windows employed for each perturbation was 17 (values for each window ranged between 0.00 and 1.00). A 2 fs time step was used, and a softcore potential was applied to keep pairwise interaction energies finite for all configurations and provide smooth free energy curves for all the simulations.^44,45^ A Langevin thermostat and a Nosé–Hoover barostat were applied for the control of the temperature and pressure, respectively. The damping coefficient for Langevin dynamics was set at 1 ps, while the barostat oscillation period was set at 100 fs and the barostat damping timescale at 50 fs. Finally, a 12 Å atom-based cutoff distance for the nonbonded interactions was used, and Coulombic interactions were handled using PME.

### TI/HREMD Simulations Using AMBER20/ff14SB

AMBER20 input files were generated using the ProFESSA workflow,^46^ which automates the setup procedure for the alchemical enhanced sampling (ACES^47^) protocol implemented in the GPU-accelerated AMBER free energy MD engine. In this protocol, a two-state simulation setup in conjunction with Hamiltonian replica-exchange MD (HREMD^48^) was employed to increase the conformational sampling of the alchemical free energy calculations.^49^ The equilibration protocol was divided into two phases; the first phase consisted of rigorous equilibration of the initial (λ = 0) and end (λ = 1) states. First, a minimization with Cartesian restraints relative to the starting structure was implemented to all nonsolvent atoms with a force constant of 5 kcal/mol/Å^2^ for 5000 steps followed by 5000 steps of minimization of the full system without any restraint. Then, the same restraints were employed for the system to perform *NPT* equilibration for 1.2 ns. Subsequently, the system was heated at a fixed volume at 310 K for 1 ns followed by 1 ns equilibration with the *NPT* ensemble. Then, annealing was performed, where the system was heated to 600 K for 0.1 ns, then simulated at 600 K for = 0.3 ns, and finally cooled down to 310 K in the last 0.4 ns. At that point, the Cartesian restraints were gradually released during a series of simulations of 0.4 ns with the *NPT* ensemble. Finally, the system was simulated with no restraints with the *NPT* ensemble for an additional 0.4 ns.

A second phase of equilibration simulations then followed, where 25 λ states were generated and equilibrated independently. The first half of the λ windows were generated from the equilibrated λ = 0 state, and the second half of the λ windows were generated from the equilibrated λ = 1 phase. The full system was minimized for 5000 steps using the steepest descent algorithm, then heated at fixed volume with 310 K for 1 ns, and equilibrated in the *NPT* ensemble for 4 ns. Finally, production runs were initiated from the final equilibrated structures, and 10 ns MD simulations were performed for each λ window using a 2 fs time step. A recently developed robust smoothstep softcore potential was applied to avoid the end-point catastrophe, particle collapse, and large gradient-jump problems often encountered in alchemical free energy simulations.^50^ For temperature control at 310 K, a Langevin thermostat was employed with a friction constant of 2.0 ps^–1^. The pressure was maintained at 1 atm using a Monte Carlo barostat (100 time steps between isotropic box scaling attempts). For the short nonbonded interactions, a 12 Å atom-based cutoff distance was used, while the long-range electrostatics were evaluated with the PME method. The AMBER20/ff14SB/thermodynamic integration (TI) protocol was performed three times using different initial velocities drawn from the Maxwell–Boltzmann distribution.

### Analysis of RBFE Calculations

To estimate the binding free energy differences from the NAMD2.14/OPLS-AA/FEP simulations, the multistate Bennett acceptance ratio (MBAR^51^) was implemented using the ParseFEP plugin^52^ of VMD 1.9.4.a. Differences in binding free energies from the AMBER20/ff14SB/TI simulations were also estimated with MBAR using Python scripts kindly provided by the Darrin York group. The reported binding free energies from the AMBER20/ff14SB/TI protocol are the mean of the three runs, while statistical uncertainties are calculated as the standard error of the mean.

## Results and Discussion

### Ligand Structural Similarity and Generation of Initial Poses

To assess the ability of BMAA and its carbamate adducts to act as GluR2 modulators, we first calculated their 2D structural similarity with respect to glutamate, which revealed a high structural similarity of the molecules under study with the natural agonist with *T*_c_ = 0.5 (see Methods for a definition of the *T*_c_) for alpha- and beta-carbamate and 0.47 for BMAA (Table S1). Moreover, we performed 3D similarity calculations of BMAA and carbamate adducts using glutamate as the query structure for the Shape Screening calculations. The results showed moderate similarity scores of beta-carbamate (0.53), alpha-carbamate (0.44), and BMAA (0.50), highlighting that beta-carbamate has the highest binding pose similarity compared to the natural agonist (Table S2).

To test whether Glide can produce valid starting structures for MD simulations, we self-docked glutamate to the GluR2 crystal structure (PDB ID 1FTJ) with Glide 6.7;^24−26^ the RMSD between the docked and the crystal glutamate poses was 1 Å (Figure S1), indicating that Glide can accurately predict the binding pose of glutamate.

As starting structures for the MD simulations of BMAA and its carbamate adducts, we used the top docked pose from the Glide docking protocol. Because BMAA has a single acidic group compared to glutamate, the acidic group could potentially bind in both directions adopting two flipped binding modes. To investigate the possibility of a flipped BMAA binding pose compared to the top Glide docked pose, we exported the first 13 poses from the Glide docking protocol. From the 13 poses, only the 7th (Δ*G* = −4.04 kcal/mol) and 8th (Δ*G* = −3.92 kcal/mol) docking poses bound the acid in the opposite direction, having a ca. 2.39 kcal/mol difference from the top docked pose (Δ*G* = −6.39 kcal/mol). Therefore, we proceeded with the most energetically favorable docking pose of BMAA and its carbamate adducts to perform the MD simulations.

### MD Simulations

We then investigated the binding of BMAA and its carbamate adducts to GluR2 by performing 100 ns equilibrium MD simulations of glutamate, BMAA, alpha-carbamate, and beta-carbamate in complex with GluR2 using two different protocols: NAMD 2.14^53^ software with the OPLS-AA force-field^27−29^ (NAMD2.14/OPLS-AA) and AMBER20^35^ software using the ff19SB^36^ and GAFF2^37^ force-fields (AMBER20/ff19SB), all in triplicates with identical starting conditions but different initial velocities. Calculation of the trajectory RMSD with respect to the initial frame of the simulation showed that all four molecules remain stable in the binding pocket of the receptor using the NAMD2.14/OPLS-AA protocol (RMSD_glutamate_ = 4.6 ± 1.3 Å, RMSD_BMAA_ = 3.3 ± 0.9 Å, RMSD_alpha-carbamate_ = 2.8 ± 0.8 Å, RMSD_beta-carbamate_ = 3.2 ± 0.8 Å) (Figure S3 and Table 1), except for BMAA in replica 1 (RMSD = 28.0 ± 18.1 Å) and beta-carbamate in replica 3 (RMSD = 11.4 ± 10.7 Å), which became solvent exposed at 27 and 45 ns, respectively. Using AMBER20/ff19SB, glutamate (RMSD = 1.7 ± 0.7 Å), alpha-carbamate (RMSD = 1.7 ± 0.2 Å), and beta-carbamate (RMSD = 2.5 ± 0.8 Å) remained stable in the binding site of the GluR2 (Figure S4 and Table 2) for all simulations. BMAA drifted off the binding site in two out of three replicas, at 74 ns in replica 1 (RMSD = 11.4 ± 15.8 Å) and at 21 ns in replica 3 (RMSD = 30.0 ± 18.4 Å). For the calculation of the average RMSD between the three replicas for both protocols, as listed in Tables 1 and 2, we did not include the RMSD values from the simulations of the dissociated molecules. The average RMSD values obtained from NAMD2.14/OPLS-AA were higher than those observed when using AMBER20/ff19SB with an average difference of ca. 1.50 Å, suggesting that OPLS-AA parameters produce more flexible simulations. Based on our results, we conclude that the substrate glutamate and the ligands alpha-carbamate and beta-carbamate are stable inside the binding pocket of the GluR2 after 100 ns, while BMAA is less stable and may dissociate from the binding site of the receptor.

**Table 1:** Average Values of the RMSD and Standard Deviation of Each Protein–Ligand Complex Were Calculated over the 100 ns MD Simulations Using NAMD2.14/OPLS-AA

replica ID	RMSD glutamate (Å)	RMSD BMAA (Å)	RMSD alpha-carbamate (Å)	RMSD beta-carbamate (Å)
1	5.9 ± 1.4	28.0 ± 18.1	3.1 ± 1.2	2.6 ± 0.3
2	3.3 ± 0.8	1.7 ± 0.3	2.8 ± 0.4	3.8 ± 1.1
3	4.7 ± 1.6	4.8 ± 1.3	2.4 ± 0.7	11.4 ± 10.7
average RMSD	4.6 ± 1.3	3.3 ± 0.9	2.8 ± 0.8	3.2 ± 0.8

**Table 2:** Average Values of RMSD and Standard Deviation of Each Protein–Ligand Complex Were Calculated over the 100 ns MD Simulations Using AMBER20/ff19SB

replica ID	RMSD glutamate (Å)	RMSD BMAA (Å)	RMSD alpha-carbamate (Å)	RMSD beta-carbamate (Å)
1	1.5 ± 0.3	11.4 ± 15.8	1.8 ± 0.3	2.5 ± 1.0
2	2.0 ± 1.0	3.0 ± 0.7	1.9 ± 0.1	2.4 ± 0.8
3	1.6 ± 0.5	30.0 ± 18.4	1.5 ± 0.2	2.6 ± 0.7
average RMSD	1.7 ± 0.7	-	1.7 ± 0.2	2.5 ± 0.8

**Figure 5:**
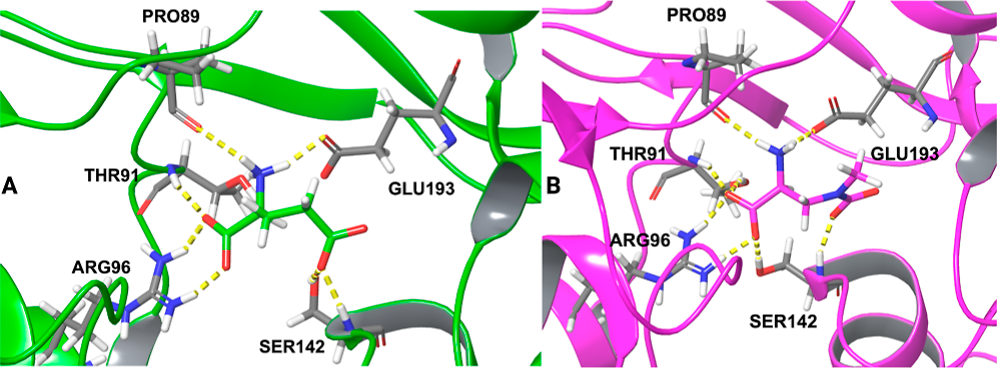
Interactions between (A) glutamate and GluR2 and (B) beta-carbamate and GluR2.

To investigate the structural basis of glutamate and beta-carbamate binding to GluR2 (beta-carbamate is the most structurally similar to glutamate (*T*_c_ = 0.50 for 2D similarity and *T*_c_ = 0.53 for 3D similarity) we used the MDTraj^54^ python package and calculated the pairwise RMSDs between the trajectory conformations to find the first cluster representative from each trajectory. The most important interactions between glutamate and GluR2, which are present in at least three replicas with both simulation protocols, are with amino acids Pro89, Thr91, Arg96, Ser142, and Glu193 (Figure 5A). Dipole−ion interactions form between the carboxylate of glutamate, the Ser142 amide group and the Ser142 side chain hydroxyl group. Glutamate also forms ion–dipole interactions with its amino group and Pro89, Thr91, and Glu193. Salt bridges are present between the glutamate charged amino group and the side chain of Glu192 as well as between the positively charged nitrogen of the guanidinium group of Arg96 and the negatively charged backbone carboxyl oxygen of glutamate. Beta-carbamate’s most important interactions with GluR2 also form with residues Pro89, Thr91, Arg96, Ser142, and Glu193 (Figure 5B). Replica 3 from the NAMD2.14/OPLS-AA protocol was not taken into consideration, as beta-carbamate dissociates from the binding site of the receptor. Beta-carbamate forms ion–dipole interactions with Pro89, Thr91, Ser142, and Glu193. A salt bridge forms between the positively charged nitrogen of the guanidinium group of Arg96 and the backbone negatively charged carboxylate of beta-carbamate. Ion–dipole interactions of glutamate with Thr91 are present across all replicas for both protocols and the corresponding beta-carbamate/Thr91 interactions, apart from the NAMD2.14/OPLS-AA replica2 simulation. The salt–bridge interaction of beta-carbamate with Arg96 is conserved in all MD simulations, whereas the glutamate/Arg96 interaction is not formed in the NAMD2.14/OPLS-AA replica2 simulation. Therefore, a comparison of the structural basis of binding to GluR2 between glutamate and beta-carbamate highlights the high similarity of the formed interactions between the two ligands.

### RBFE Calculations

The RBFE of beta-carbamate with respect to the natural agonist glutamate in GluR2 was determined by alchemical free energy calculations (Figure 3). We examined beta-carbamate because it is the most structurally similar to glutamate (*T*_c_ = 0.50 for 2D similarity and *T*_c_ = 0.53 for 3D similarity) and remained bound in the binding site of GluR2 during the MD simulations. Prior to the RBFE calculations, we examined whether the beta-carbamate could adopt multiple binding modes within the pocket of GluR2 using Glide docking calculations. We exported 16 docking poses and observed that the 3rd (Δ*G* = −7.35 kcal/mol), 8th (Δ*G* = −5.75 kcal/mol), and 10th (Δ*G* = −5.20 kcal/mol) docking poses flip with respect to the glutamate PDB ID1FTJ pose. The flipped binding pose with the most favorable free energy had a 1.27 kcal/mol difference from the dominant binding pose (Δ*G* = −8.62 kcal/mol). Therefore, the flipping of beta-carbamate during the course of the RBFE calculations should be carefully considered. We thus performed two different protocols: FEP coupled with MD simulations using the NAMD 2.14^53,55^ software and the OPLS-AA^27−29^ force-field (NAMD2.14/OPLS-AA/FEP) and TI coupled with MD simulation using the AMBER20^35,56^ software, the ff14SB^57^ force-field for the protein, and the GAFF2^37^ force-field for the ligands (AMBER20/ff14SB/TI). The latter protocol uses HREMD that accounts for flipping of the molecules during the calculations. The ACES protocol implemented in the GPU-accelerated AMBER free energy MD engine has been successfully tested in complex protein–ligand systems that involve ring flips (CDK2) and concerted ligand and protein side-chain conformational changes (T4-lysozyme).^47^ Thus, the AMBER20/ff14SB/TI method may account for a potential flipped binding mode of the beta-carbamate adduct. In addition, AMBER TI RBFE calculations have also been successfully performed in charged ligand perturbations. In the framework of the 2018 Drug Design Data Resource (D3R) grand challenge 4, all the predicted relative binding free energies of the Cathepsin S data set were within 2 kcal/mol error compared to the experimental results.^58^ The authors concluded that the high accuracy of the predictions resulted from the force field parameters (ff14SB and GAFF); these are also employed in our AMBER20/ff14SB/TI protocol. In addition, RBFE calculations using the same AMBER force fields and TI calculations were also performed in the PTP1B data set and showed an overall mean unsigned error of 1.6 kcal/mol.^59^ This is of critical importance as the binding energetics for glutamate as well as carbamate adducts appear to be primarily driven by electrostatics, and atomistic force fields tend to overly reward charge interactions.^60^ Our AMBER20/ff14SB/TI protocol was repeated two more times to ensure reproducibility of the results.

We initially introduced one intermediate molecule ("intermediate 1") to connect glutamate and the beta-carbamate adduct of BMAA to increase the phase space overlap so as to obtain a more accurate estimate of the relative binding free energy and to close the thermodynamic cycle and measure the hysteresis of the calculation, thus monitoring the convergence of the RBFE calculation. The perturbation paths formed a closed cycle between the calculated molecules that was used to monitor the convergence of our RBFE calculations (Figure S5). In a converged alchemical free energy calculation, the sum of the free energies over the closed cycle should be zero or close to zero. However, simulations with NAMD2.14/OPLS-AA/FEP resulted in a cycle closure error of −2.11 ± 0.27 kcal/mol. The phase space overlaps between the λ states are listed in Table S3. To overcome this issue, we introduced additional λ windows for each perturbation, where the convergence between neighboring λ windows was clearly low (overlap <40%, see Supporting Information). The addition of λ windows increased the average phase space overlap of the perturbation glutamate-intermediate1, by 27.1% (from 33% in the λ windows with overlap <40% it increased to 59.7%), for intermediate1-beta carbamate, the overlap increased 29.5% (from 30.30% in the λ windows with overlap <40%, it increased to 59.9%), and for the beta carbamate - > glutamate perturbation it increased 37.2% (from 21.27% in the λ windows with overlap <40%, it increased to 64.41%), respectively (see also the Supporting Information for each perturbation). However, even if the phase space overlap between neighboring windows increased, the cycled closed error changed by only 0.1 kcal/mol. To reduce the cycle closure error and to increase the phase space overlap between the two ligands, we added another intermediate molecule, "intermediate-2" (Figure 4). Although we did not gain a clear benefit in increasing the phase space overlap from adding the second intermediate molecule (the phase space overlap improved in some neighboring λ windows but deteriorated in others. Particularly, others, see Supporting Information "Results" section for details), the cycle closure error improved significantly to 1.10 ± 0.09 kcal/mol, compared to the initial three-state RBFE calculations (−2.11 ± 0.27 kcal/mol). Therefore, adding the second intermediate molecule was needed to increase the convergence of the calculation. We thus employed the same scheme in the AMBER20/ff14SB/TI protocol, resulting in a cycle closure error equal to −0.57 ± 0.41 kcal/mol.

To assess the free energy convergence of our calculations, we measured the degree of overlap between the probability distributions characterizing the equilibrium ensembles for the forward and backward calculations. The phase space overlap between the λ states ranged between 17.7 and 90.9% using NAMD2.14/OPLS-AA/FEP (Figures S6, S8, S10, S12, S14, S16, S18 and S20 and Table S4) and between 74.5 and 98.3% using AMBER20/ff14SB/TI, except for the transition from the initial state to the next neighboring state (λ_0→1_), which refers to the intermediate-1 → beta-carbamate. In this case, the overlap was 21.3, 21.2 and 21.2% for replicas 1, 2 and 3, respectively, in the solvent and 22.8, 19.7 and 20.0% for replicas 1, 2 and 3, respectively, in the complex system (Figures S6, S8, S10, S12, S14, S16, S18 and S20 and Table S5–S7), suggesting that more neighboring λ windows could be added in this transformation.

Free energy (Δ*G*) versus λ for the NAMD2.14/OPLS-AA/FEP protocol and derivative of the gradient of the potential energy (⟨∂U/∂λ⟩) as a function of λ versus λ for the AMBER20/ff14SB/TI protocol were also used to determine regions of high curvature across the intermediate λ windows (Figures S7, S9, S11, S13, S15, S17, S19 and S21). These regions of phase space serve as a helpful diagnostic as they denote that more sampling or more dense lambda spacing is required to reduce the error of the free energy estimate. In our simulations, we observed low curvature sets of Δ*G* or ⟨∂U/∂λ⟩ sets, with the possible exception of the λ_0→1_ transition belonging to the intermediate-1 → beta-carbamate for both solvent and complex phases. Thus, a good practice would be to ensure a denser λ spacing in this area.

Overall, our results indicate that the beta-carbamate adduct of BMAA is more potent compared to the natural agonist: the difference in free energy of binding (ΔΔ*G*) of beta-carbamate with respect to glutamate is equal to −1.61 ± 0.33 kcal/mol using the NAMD2.14/OPLS-AA/FEP protocol and −2.77 ± 0.12 kcal/mol using the AMBER20/ff14SB/TI protocol (Table 3). Therefore, beta-carbamate could potentially bind to GluR2 more strongly than glutamate, potentially leading to dysfunction in neurons and to neurodegenerative diseases. Finally, both protocols showed comparable binding affinities for each perturbation. The bigger differences in binding free energies observed for glutamate → intermediate 1 (1.34 kcal/mol) and beta-carbamate → intermediate-2 (1.39 kcal/mol) perturbations can be attributed to the error of the method, which has been determined to be between 1.5 and 2 kcal/mol, or from the different settings (e.g., force-fields, sampling technique) applied in the two protocols.^41^

**Table 3:** Binding Free Energy Differences for Each Perturbation and the Cycle Closure Error Using NAMD2.14/OPLS-AA/FEP and AMBER20/ff14SB/TI

	NAMD2.14/OPLS-AA/FEP	AMBER20/ff14SB/TI
perturbation	ΔΔ*G* (kcal/mol)	ΔΔ*G* (kcal/mol)
glutamate → intermediate-1	1.04 ± 0.08	–0.30 ± 0.12
intermediate-1 → beta-carbamate	–1.54 ± 0.06	–2.47 ± 0.03
beta-carbamate → intermediate-2	2.36 ± 0.14	0.97 ± 0.35
intermediate-2 → glutamate	–0.75 ± 0.08	1.23 ± 0.19
cycle closure error	1.10 ± 0.09	–0.57 ± 0.41

## Conclusions

BMAA has been linked to multiple neurodegenerative diseases, but its disease-promoting mechanism remains unknown. BMAA exhibits neurotoxic activity only in the presence of HCO_3_^–^ ions, which leads to the formation of carbamates that share high structural similarity with glutamic acid. Thus, it has been hypothesized that BMAA and/or its carbamate adducts contribute to neurodegenerative diseases by binding to the glutamate binding site of GluR2.^3^ Although here we do not attempt to describe the disease-promoting mechanism of BMAA, we provide insights into whether BMAA and/or its carbamate adducts bind on GluR2, which could potentially modulate GluR2 at its glutamate binding site and possibly contribute to neurodegenerative diseases.

While the differences between BMAA and its carbamate adducts may look structurally trivial, it is known in the medicinal chemistry literature that small changes in ligand structure may lead to large changes in ligand–protein affinity. Medicinal chemists have long been familiar with the “magic methyl” effect, which is the dramatic change in affinity that may be observed by adding a single methyl group.^61^ Additions of fluorine, nitrogen, and other atoms may give rise to the same effect. Because the small changes between glutamate, BMAA, and its carbamate adducts cannot be quantified in terms of their affinity for the GluR2 by simply eyeballing the structure, the application of robust and accurate methodologies is needed to calculate the affinity of glutamate compared to its adducts on GluR2, in addition to experimental results to determine these effects.

To investigate whether BMAA and/or its adducts bind on GluR2, we first performed equilibrium MD simulations to monitor the stability of glutamate, BMAA, and two carbamate adducts on the GluR2 in atomic-level detail using two different protocols, NAMD2.14/OPLS-AA and AMBER20/ff19SB. Our findings show that the substrate glutamate and the ligands alpha-carbamate and beta-carbamate remain stable in the binding pocket of the GluR2 for 100 ns, while BMAA is less stable and dissociates from the receptor in 50% of the simulations. This observation is in agreement with experimental assays showing that BMAA was nontoxic in the absence of bicarbonate.^3^ Moreover, glutamate and beta-carbamate share similar interactions in the binding pocket of GluR2, possibly due to their high structural similarity.

Next, to compute the difference in the free energy of binding between the beta-carbamate adduct of BMAA and glutamate to GluR2, we performed alchemical free energy calculations with two different protocols, NAMD2.14/OPLS-AA/FEP and AMBER20/ff14SB/TI. We did not investigate the binding of BMAA to GluR2 using RBFE calculations because the total charge of these molecules within the perturbation would not remain conserved. Changing the total system charge could yield accurate results,^62^ but there are limited successful examples of charge changes during RBFE calculations in the literature.^41^

Results from the RBFE calculations between the beta-carbamate adduct of BMAA and glutamate binding to GluR2 reveal that using two intermediate molecules instead of one, which was the original setup, significantly improved the closed cycle error of the simulations. The phase space overlap between the different λ states ranged between 17.7 and 90.9% with NAMD2.14/OPLS-AA/FEP and between 74.5 and 98.3% using AMBER20/ff14SB/TI, indicating that the latter protocol was more efficient for this study. In both cases, the difference in the free energy of binding between glutamate and beta-carbamate was negative (−1.61 ± 0.33 kcal/mol for the NAMD2.14/OPLS-AA/FEP protocol and −2.77 ± 0.12 kcal/mol for the AMBER20/ff14SB/TI protocol). Although these values are close to the error of the method, they do suggest that beta-carbamate has a comparable, if not stronger, binding affinity to GluR2 compared to glutamate. Therefore, based on RBFE calculations and studying the interactions between GluR2 and BMAA and its adducts, we provide a rationale that BMAA carbamate adducts may be in fact the modulators of GluR2 and not BMAA itself.

This study also employs two different MD packages and force-fields to ensure the consistency of the results across the codes. Both MD protocols were able to predict the stability of glutamate, alpha-carbamate, and beta-carbamate inside the binding pocket of the GluR2 compared to BMAA. The average RMSD values obtained from NAMD2.14/OPLS-AA were ca. 1.50 Å higher than those observed when using the AMBER20/ff19SB protocol. In addition, the free energy results obtained from the two protocols were also comparable within the limits of the error of the method (1.16 kcal/mol). However, AMBER20/ff14SB/TI was more efficient for this study based on the phase space overlap between the λ neighbors compared to the NAMD2.14/OPLS-AA/FEP protocol. In addition, the ProFESSA workflow made AMBER/TI easier to use compared to the NAMD/FEP setup.

Although alchemical free energy calculations are now routinely used in the pharmaceutical industry in lead optimization cycles, they are still not streamlined with noncommercial software. In this paper, we report issues that we faced during the calculations concerning convergence issues and how we solved them (introduction of two intermediate molecules and monitoring the hysteresis of the calculation). We conducted a technically sound study using two different alchemical free energy protocols, and given the high interest of the pharmaceutical industry in alchemical free energy calculations, we strongly believe that such studies are important to be demonstrated in the current literature. Especially for the AMBER20/ff14SB/TI protocol, we employed a state-of-the-art methodology that was published recently,^46,47,49^ we tested it, and provided a proof of concept for its use. Finally, we provide the full data set for this protocol, as well as for all the other MD and RBFE protocols that we applied.

Overall, both MD protocols highlight the stability of the beta-carbamate/GluR2 complex, while both RBFE protocols predict a comparable binding affinity of beta-carbamate and natural agonist glutamate. Therefore, the beta-carbamate adduct of BMAA could potentially bind equipotently or more strongly to GluR2 compared to glutamate and may provoke dysfunction in neurons, leading to neurodegenerative diseases; this hypothesis should be further examined through experimental assays.

## Data Availability

The NAMD 2.14 software package is available at https://www.ks.uiuc.edu/Research/namd/and the AMBER20 software package is available at http://ambermd.org/. FEPrepare Web server is freely accessible at https://feprepare.vi-seem.eu/and the LigParGen Web server is freely accessible at http://zarbi.chem.yale.edu/ligpargen/. The input/output files generated during this study are available at 10.5281/zenodo.7730768.

## Supporting Information Available

The ligands’ similarity with glutamate, model construction, RBFE calculations’ theory and results concerning self-docking, and alchemical free energy calculations starting structures and results (PDF)

## References

[ref1] Vyas K. J.; Weiss J. H. BMAA - an Unusual Cyanobacterial Neurotoxin. Amyotroph Lateral Scler. 2009, 10 (sup2), 50–55. 10.3109/17482960903268742. 19929732

[ref2] Porojan C.; Mitrovic S. M.; Yeo D. C. J.; Furey A. Overview of the potent cyanobacterial neurotoxin β-methylamino-L-alanine (BMAA) and its analytical determination. Food Addit. Contam. 2016, 33 (10), 1570–1586. 10.1080/19440049.2016.1217070. 27652898

[ref3] Diaz-parga P.; Goto J. J.; Krishnan V. V. Chemistry and Chemical Equilibrium Dynamics of BMAA and Its Carbamate Adducts. Neurotox. Res. 2018, 33 (1), 76–86. 10.1007/s12640-017-9801-2. 28921378 PMC5834315

[ref4] Myers T. G.; Nelson S. D. Neuroactive Carbamate Adducts of Beta-N-Methylamino-L-Alanine and Ethylenediamine. Detection and Quantitation under Physiological Conditions by 13C NMR. J. Biol. Chem. 1990, 265 (18), 10193–10195. 10.1016/S0021-9258(18)86928-9. 2113048

[ref5] Catarzi D.; Colotta V.; Varano F. Competitive Gly/NMDA Receptor Antagonists. Curr. Top. Med. Chem. 2006, 6 (8), 809–821. 10.2174/156802606777057544. 16719819

[ref6] Mayer M. L. Crystal Structures of the GluR5 and GluR6 Ligand Binding Cores: Molecular Mechanisms Underlying Kainate Receptor Selectivity. Neuron 2005, 45 (4), 539–552. 10.1016/j.neuron.2005.01.031. 15721240

[ref7] Zhu S.; Gouaux E. Structure and Symmetry Inform Gating Principles of Ionotropic Glutamate Receptors. Neuropharmacology 2017, 112 (Pt A), 11–15. 10.1016/j.neuropharm.2016.08.034. 27663701 PMC5082733

[ref8] Allen C. N.; Spencer P. S.; Carpenter D. O. β-N-Methylamino-l-alanine in the presence of bicarbonate is an agonist at non- N-methyl-d-aspartate-type receptors. Neuroscience 1993, 54 (3), 567–574. 10.1016/0306-4522(93)90228-8. 7687332

[ref9] Goto J. J.; Koenig J. H.; Ikeda K. The physiological effect of ingested β-N-methylamino-L-alanine on a glutamatergic synapse in an in vivo preparation. Comp. Biochem. Physiol. C Toxicol. Pharmacol. 2012, 156 (3–4), 171–177. 10.1016/j.cbpc.2012.07.004. 22841708

[ref10] Isaac J. T. R.; Ashby M. C.; McBain C. J. The Role of the GluR2 Subunit in AMPA Receptor Function and Synaptic Plasticity. Neuron 2007, 54 (6), 859–871. 10.1016/j.neuron.2007.06.001. 17582328

[ref11] Ahmed A. H.; Wang Q.; Sondermann H.; Oswald R. E. Structure of the S1S2 Glutamate Binding Domain of GLuR3. Proteins: Struct., Funct., Bioinf. 2009, 75 (3), 628–637. 10.1002/prot.22274. PMC2732992 19003990

[ref12] Armstrong N.; Gouaux E. Mechanisms for Activation and Antagonism of an AMPA-Sensitive Glutamate Receptor: Crystal Structures of the GluR2 Ligand Binding Core. Neuron 2000, 28 (1), 165–181. 10.1016/S0896-6273(00)00094-5. 11086992

[ref13] Furukawa H.; Singh S. K.; Mancusso R.; Gouaux E. Subunit Arrangement and Function in NMDA Receptors. Nature 2005, 438 (7065), 185–192. 10.1038/nature04089. 16281028

[ref63] Speranskiy K.; Kurnikova M. On the Binding Determinants of the Glutamate Agonist with the Glutamate Receptor Ligand Binding Domain. Biochemistry 2005, 44 (34), 11508–11517. 10.1021/bi050547w. 16114887

[ref64] Mamonova T.; Yonkunas M. J.; Kurnikova M. G. Energetics of the Cleft Closing Transition and the Role of Electrostatic Interactions in Conformational Rearrangements of the Glutamate Receptor Ligand Binding Domain. Biochemistry 2008, 47 (42), 11077–11085. 10.1021/bi801367d. 18823129 PMC2814871

[ref14] Yonkunas M.; Buddhadev M.; Flores Canales J. C.; Kurnikova M. G. Configurational Preference of the Glutamate Receptor Ligand Binding Domain Dimers. Biophys. J. 2017, 112 (11), 2291–2300. 10.1016/j.bpj.2017.04.042. 28591602 PMC5474840

[ref15] Yu A.; Lau A. Y. Energetics of Glutamate Binding to an Ionotropic Glutamate Receptor. J. Phys. Chem. B 2017, 121 (46), 10436–10442. 10.1021/acs.jpcb.7b06862. 29065265 PMC5716343

[ref16] Heinzelmann G.; Chen P.-C.; Kuyucak S. Computation of Standard Binding Free Energies of Polar and Charged Ligands to the Glutamate Receptor GluA2. J. Phys. Chem. B 2014, 118 (7), 1813–1824. 10.1021/jp412195m. 24479628

[ref17] Bajusz D.; Rácz A.; Héberger K. Why Is Tanimoto Index an Appropriate Choice for Fingerprint-Based Similarity Calculations?. J. Cheminf. 2015, 7 (1), 20 10.1186/s13321-015-0069-3. PMC4456712 26052348

[ref18] Karatzas E.; Zamora J. E.; Athanasiadis E.; Dellis D.; Cournia Z.; Spyrou G. M. ChemBioServer 2.0: An Advanced Web Server for Filtering, Clustering and Networking of Chemical Compounds Facilitating Both Drug Discovery and Repurposing. Bioinformatics 2020, 36 (8), 2602–2604. 10.1093/bioinformatics/btz976. 31913451 PMC7178400

[ref19] Maestro, Schrödinger, LLC. Schrödinger Release 2023–1; Maestro, Schrödinger, LLC: New York, NY, 2023.

[ref20] Dixon S. L.; Smondyrev A. M.; Rao S. N. PHASE: A Novel Approach to Pharmacophore Modeling and 3D Database Searching. Chem. Biol. Drug Des. 2006, 67 (5), 370–372. 10.1111/j.1747-0285.2006.00384.x. 16784462

[ref21] Dixon S. L.; Smondyrev A. M.; Knoll E. H.; Rao S. N.; Shaw D. E.; Friesner R. A. PHASE: A New Engine for Pharmacophore Perception, 3D QSAR Model Development, and 3D Database Screening: 1. Methodology and Preliminary Results. J. Comput. Aided Mol. Des. 2006, 20 (10–11), 647–671. 10.1007/s10822-006-9087-6. 17124629

[ref22] Lionta E.; Spyrou G.; Vassilatis D.; Cournia Z. Structure-Based Virtual Screening for Drug Discovery: Principles, Applications and Recent Advances. Curr. Top. Med. Chem. 2014, 14 (16), 1923–1938. 10.2174/1568026614666140929124445. 25262799 PMC4443793

[ref23] Altschul S. F.; Gish W.; Miller W.; Myers E. W.; Lipman D. J. Basic Local Alignment Search Tool. J. Mol. Biol. 1990, 215 (3), 403–410. 10.1016/S0022-2836(05)80360-2. 2231712

[ref24] Friesner R. A.; Murphy R. B.; Repasky M. P.; Frye L. L.; Greenwood J. R.; Halgren T. A.; Sanschagrin P. C.; Mainz D. T. Extra Precision Glide: Docking and Scoring Incorporating a Model of Hydrophobic Enclosure for Protein-Ligand Complexes. J. Med. Chem. 2006, 49 (21), 6177–6196. 10.1021/jm051256o. 17034125

[ref25] Friesner R. A.; Banks J. L.; Murphy R. B.; Halgren T. A.; Klicic J. J.; Mainz D. T.; Repasky M. P.; Knoll E. H.; Shelley M.; Perry J. K.; Shaw D. E.; Francis P.; Shenkin P. S. Glide: A New Approach for Rapid, Accurate Docking and Scoring. 1. Method and Assessment of Docking Accuracy. J. Med. Chem. 2004, 47 (7), 1739–1749. 10.1021/jm0306430. 15027865

[ref26] Halgren T. A.; Murphy R. B.; Friesner R. A.; Beard H. S.; Frye L. L.; Pollard W. T.; Banks J. L. Glide: A New Approach for Rapid, Accurate Docking and Scoring. 2. Enrichment Factors in Database Screening. J. Med. Chem. 2004, 47 (7), 1750–1759. 10.1021/jm030644s. 15027866

[ref27] Robertson M. J.; Tirado-Rives J.; Jorgensen W. L. Improved Peptide and Protein Torsional Energetics with the OPLS-AA Force Field. J. Chem. Theory Comput. 2015, 11 (7), 3499–3509. 10.1021/acs.jctc.5b00356. 26190950 PMC4504185

[ref28] Dodda L. S.; de Vaca I. C.; Tirado-Rives J.; Jorgensen W. L. LigParGen Web Server: An Automatic OPLS-AA Parameter Generator for Organic Ligands. Nucleic Acids Res. 2017, 45 (W1), W331–W336. 10.1093/nar/gkx312. 28444340 PMC5793816

[ref29] Jorgensen W. L.; Tirado-Rives J. Potential Energy Functions for Atomic-Level Simulations of Water and Organic and Biomolecular Systems. Proc. Natl. Acad. Sci. U.S.A. 2005, 102 (19), 6665–6670. 10.1073/pnas.0408037102. 15870211 PMC1100738

[ref30] Jorgensen W. L.; Chandrasekhar J.; Madura J. D.; Impey R. W.; Klein M. L. Comparison of Simple Potential Functions for Simulating Liquid Water. J. Chem. Phys. 1983, 79 (2), 926–935. 10.1063/1.445869.

[ref31] Darden T.; York D.; Pedersen L. Particle mesh Ewald: An N·log(N) method for Ewald sums in large systems. J. Chem. Phys. 1993, 98 (12), 10089–10092. 10.1063/1.464397.

[ref32] Davidchack R. L.; Handel R.; Tretyakov M. V. Langevin Thermostat for Rigid Body Dynamics. J. Chem. Phys. 2009, 130 (23), 234101 10.1063/1.3149788. 19548705

[ref33] Martyna G. J.; Tobias D. J.; Klein M. L. Constant Pressure Molecular Dynamics Algorithms. J. Chem. Phys. 1994, 101 (5), 4177–4189. 10.1063/1.467468.

[ref34] Humphrey W.; Dalke A.; Schulten K. VMD: Visual Molecular Dynamics. J. Mol. Graph. 1996, 14 (1), 33–38. 10.1016/0263-7855(96)00018-5. 8744570

[ref35] Case D. A.; Belfon K.; Ben-Shalom I. Y.; Brozell S. R.; Cerutti D. S.; Cheatham T. E. III, Cruzeiro V. W. D.; Darden T. A.; Duke R. E.; Giambasu G.; Gilson M. K.; Gohlke H.; Goetz A. W., Harris R.; Izadi S.; Iz-Mailov S. A., Kasavajhala K.; Kovalenko A.; Krasny R.; Kurtzman T.; Lee T. S.; LeGrand S.; Li P.; Lin C.; Liu J.; Luchko T.; Luo R.; Man V.; Merz K. M.; Miao Y.; Mikhailovskii O.; Monard G.; Nguyen H.; Onufriev A.; Pan F.; Pantano S.; Qi R.; Roe D. R.; Roitberg A.; Sagui C.; Schott-Verdugo S.; Shen J.; Simmerling C. L.; Skrynnikov N. R.; Smith J.; Swails J.; Walker R. C.; Wang J.; Wilson L.; Wolf R. M.; Wu X.; Xiong Y.; Xue Y.; York D. M.; Kollman P. A. AMBER 2020; University of California: San Francisco, 2020.

[ref36] Tian C.; Kasavajhala K.; Belfon K. A. A.; Raguette L.; Huang H.; Migues A. N.; Bickel J.; Wang Y.; Pincay J.; Wu Q.; Simmerling C. ff19SB: Amino-Acid-Specific Protein Backbone Parameters Trained against Quantum Mechanics Energy Surfaces in Solution. J. Chem. Theory Comput. 2020, 16 (1), 528–552. 10.1021/acs.jctc.9b00591. 31714766 PMC13071887

[ref37] Wang J.; Wolf R. M.; Caldwell J. W.; Kollman P. A.; Case D. A. Development and Testing of a General Amber Force Field. J. Comput. Chem. 2004, 25 (9), 1157–1174. 10.1002/jcc.20035. 15116359

[ref38] Jakalian A.; Jack D. B.; Bayly C. I. Fast, efficient generation of high-quality atomic charges. AM1-BCC model: II. Parameterization and validation. J. Comput. Chem. 2002, 23 (16), 1623–1641. 10.1002/jcc.10128. 12395429

[ref39] Faller R.; de Pablo J. J. Constant Pressure Hybrid Molecular Dynamics-Monte Carlo Simulations. J. Chem. Phys. 2002, 116 (1), 55–59. 10.1063/1.1420460.

[ref40] Zwanzig R. W. High-Temperature Equation of State by a Perturbation Method. I. Nonpolar Gases. J. Chem. Phys. 1954, 22 (8), 1420–1426. 10.1063/1.1740409.

[ref41] Cournia Z.; Allen B.; Sherman W. Relative Binding Free Energy Calculations in Drug Discovery: Recent Advances and Practical Considerations. J. Chem. Inf. Model. 2017, 57 (12), 2911–2937. 10.1021/acs.jcim.7b00564. 29243483

[ref42] Athanasiou C.; Vasilakaki S.; Dellis D.; Cournia Z. Using Physics-Based Pose Predictions and Free Energy Perturbation Calculations to Predict Binding Poses and Relative Binding Affinities for FXR Ligands in the D3R Grand Challenge 2. J. Comput. Aided Mol. Des. 2018, 32 (1), 21–44. 10.1007/s10822-017-0075-9. 29119352

[ref43] Zavitsanou S.; Tsengenes A.; Papadourakis M.; Amendola G.; Chatzigoulas A.; Dellis D.; Cosconati S.; Cournia Z. FEPrepare: A Web-Based Tool for Automating the Setup of Relative Binding Free Energy Calculations. J. Chem. Inf. Model. 2021, 61 (9), 4131–4138. 10.1021/acs.jcim.1c00215. 34519200

[ref44] Beutler T. C.; Mark A. E.; van Schaik R. C.; Gerber P. R.; van Gunsteren W. F. Avoiding Singularities and Numerical Instabilities in Free Energy Calculations Based on Molecular Simulations. Chem. Phys. Lett. 1994, 222 (6), 529–539. 10.1016/0009-2614(94)00397-1.

[ref45] Zacharias M.; Straatsma T. P.; McCammon J. A. Separation-shifted Scaling, a New Scaling Method for Lennard-Jones Interactions in Thermodynamic Integration. J. Chem. Phys. 1994, 100 (12), 9025–9031. 10.1063/1.466707.

[ref46] Ganguly A.; Tsai H.-C.; Fernández-Pendás M.; Lee T.-S.; Giese T. J.; York D. M. AMBER Drug Discovery Boost Tools: Automated Workflow for Production Free-Energy Simulation Setup and Analysis (ProFESSA). J. Chem. Inf. Model. 2022, 62 (23), 6069–6083. 10.1021/acs.jcim.2c00879. 36450130 PMC9881431

[ref47] Lee T.-S.; Tsai H.-C.; Ganguly A.; York D. M. ACES: Optimized Alchemically Enhanced Sampling. J. Chem. Theory Comput. 2023, 19, 472–487. 10.1021/acs.jctc.2c00697. PMC10333454 36630672

[ref48] Meng Y.; Dashti D. S.; Roitberg A. E. Computing Alchemical Free Energy Differences with Hamiltonian Replica Exchange Molecular Dynamics (H-REMD) Simulations. J. Chem. Theory Comput. 2011, 7 (9), 2721–2727. 10.1021/ct200153u. 22125475 PMC3223983

[ref49] Tsai H.-C.; Lee T.-S.; Ganguly A.; Giese T. J.; Ebert M. C.; Labute P.; Merz K. M.; York D. M. AMBER Free Energy Tools: A New Framework for the Design of Optimized Alchemical Transformation Pathways. J. Chem. Theory Comput. 2023, 19, 640–658. 10.1021/acs.jctc.2c00725. PMC10329732 36622640

[ref50] Lee T.-S.; Lin Z.; Allen B. K.; Lin C.; Radak B. K.; Tao Y.; Tsai H.-C.; Sherman W.; York D. M. Improved Alchemical Free Energy Calculations with Optimized Smoothstep Softcore Potentials. J. Chem. Theory Comput. 2020, 16 (9), 5512–5525. 10.1021/acs.jctc.0c00237. 32672455 PMC7494069

[ref51] Shirts M. R.; Chodera J. D. Statistically Optimal Analysis of Samples from Multiple Equilibrium States. J. Chem. Phys. 2008, 129 (12), 124105 10.1063/1.2978177. 19045004 PMC2671659

[ref52] Liu P.; Dehez F.; Cai W.; Chipot C. A Toolkit for the Analysis of Free-Energy Perturbation Calculations. J. Chem. Theory Comput. 2012, 8 (8), 2606–2616. 10.1021/ct300242f. 26592106

[ref53] Phillips J. C.; Braun R.; Wang W.; Gumbart J.; Tajkhorshid E.; Villa E.; Chipot C.; Skeel R. D.; Kalé L.; Schulten K. Scalable Molecular Dynamics with NAMD. J. Comput. Chem. 2005, 26 (16), 1781–1802. 10.1002/jcc.20289. 16222654 PMC2486339

[ref54] McGibbon R. T.; Beauchamp K. A.; Harrigan M. P.; Klein C.; Swails J. M.; Hernández C.; Schwantes C. R.; Wang L.-P.; Lane T. J.; Pande V. S. MDTraj: A Modern Open Library for the Analysis of Molecular Dynamics Trajectories. Biophys. J. 2015, 109 (8), 1528–1532. 10.1016/j.bpj.2015.08.015. 26488642 PMC4623899

[ref55] Jiang W.; Chipot C.; Roux B. Computing Relative Binding Affinity of Ligands to Receptor: An Effective Hybrid Single-Dual-Topology Free-Energy Perturbation Approach in NAMD. J. Chem. Inf. Model. 2019, 59 (9), 3794–3802. 10.1021/acs.jcim.9b00362. 31411473 PMC7007809

[ref56] Lee T.-S.; Allen B. K.; Giese T. J.; Guo Z.; Li P.; Lin C.; McGee T. D. Jr.; Pearlman D. A.; Radak B. K.; Tao Y.; Tsai H.-C.; Xu H.; Sherman W.; York D. M. Alchemical Binding Free Energy Calculations in AMBER20: Advances and Best Practices for Drug Discovery. J. Chem. Inf. Model. 2020, 60 (11), 5595–5623. 10.1021/acs.jcim.0c00613. 32936637 PMC7686026

[ref57] Maier J. A.; Martinez C.; Kasavajhala K.; Wickstrom L.; Hauser K. E.; Simmerling C. ff14SB: Improving the Accuracy of Protein Side Chain and Backbone Parameters from ff99SB. J. Chem. Theory Comput. 2015, 11 (8), 3696–3713. 10.1021/acs.jctc.5b00255. 26574453 PMC4821407

[ref58] Zou J.; Tian C.; Simmerling C. Blinded Prediction of Protein-Ligand Binding Affinity Using Amber Thermodynamic Integration for the 2018 D3R Grand Challenge 4. J. Comput. Aided Mol. Des. 2019, 33 (12), 1021–1029. 10.1007/s10822-019-00223-x. 31555923 PMC6899192

[ref59] Song L. F.; Lee T.-S.; Zhu C.; York D. M.; Merz K. M. Jr Using AMBER18 for Relative Free Energy Calculations. J. Chem. Inf. Model. 2019, 59 (7), 3128–3135. 10.1021/acs.jcim.9b00105. 31244091 PMC7371000

[ref60] Ahmed M. C.; Papaleo E.; Lindorff-Larsen K. How Well Do Force Fields Capture the Strength of Salt Bridges in Proteins?. PeerJ 2018, 6, e4967 10.7717/peerj.4967. 29910983 PMC6001725

[ref61] Schönherr H.; Cernak T. Profound Methyl Effects in Drug Discovery and a Call for New C–H Methylation Reactions. Angew. Chem., Int. Ed. 2013, 52 (47), 12256–12267. 10.1002/anie.201303207. 24151256

[ref62] Zhou R.; Das P.; Royyuru A. K. Single Mutation Induced H3N2 Hemagglutinin Antibody Neutralization: A Free Energy Perturbation Study. J. Phys. Chem. B 2008, 112 (49), 15813–15820. 10.1021/jp805529z. 19367871

